# Distributed usability evaluation of the Pennsylvania Cancer Atlas

**DOI:** 10.1186/1476-072X-7-36

**Published:** 2008-07-11

**Authors:** Tanuka Bhowmick, Anthony C Robinson, Adrienne Gruver, Alan M MacEachren, Eugene J Lengerich

**Affiliations:** 1 GeoVISTA Center, Department of Geography, The Pennsylvania State University, University Park, USA; 2 Department of Public Health Sciences, Penn State College of Medicine, Hershey, USA; 3 Penn State Hershey Cancer Institute, Hershey, USA

## Abstract

**Background:**

The Pennsylvania Cancer Atlas (PA-CA) is an interactive online atlas to help policy-makers, program managers, and epidemiologists with tasks related to cancer prevention and control. The PA-CA includes maps, graphs, tables, that are dynamically linked to support data exploration and decision-making with spatio-temporal cancer data. Our Atlas development process follows a user-centered design approach. To assess the usability of the initial versions of the PA-CA, we developed and applied a novel strategy for soliciting user feedback through multiple distributed focus groups and surveys. Our process of acquiring user feedback leverages an online web application (e-Delphi). In this paper we describe the PA-CA, detail how we have adapted e-Delphi web application to support usability and utility evaluation of the PA-CA, and present the results of our evaluation.

**Results:**

We report results from four sets of users. Each group provided structured individual and group assessments of the PA-CA as well as input on the kinds of users and applications for which it is best suited. Overall reactions to the PA-CA are quite positive. Participants did, however, provide a range of useful suggestions. Key suggestions focused on improving interaction functions, enhancing methods of temporal analysis, addressing data issues, and providing additional data displays and help functions. These suggestions were incorporated in each design and implementation iteration for the PA-CA and used to inform a set of web-atlas design principles.

**Conclusion:**

For the Atlas, we find that a design that utilizes linked map, graph, and table views is understandable to and perceived to be useful by the target audience of cancer prevention and control professionals. However, it is clear that considerable variation in experience using maps and graphics exists and for those with less experience, integrated tutorials and help features are needed. In relation to our usability assessment strategy, we find that our distributed, web-based method for soliciting user input is generally effective. Advantages include the ability to gather information from users distributed in time and space and the relative anonymity of the participants while disadvantages include less control over when and how often participants provide input and challenges for obtaining rich input.

## Background

### Introduction and Motivation

The paper has two goals. First, it introduces a new, web-based cancer atlas designed to support state-level comprehensive cancer control activities. Second, it introduces a novel strategy for obtaining individual and collaborative input from distributed individuals as part of a user-centered design process. Each is outlined below briefly.

Cancer is the second leading cause of death in the United States, with approximately 30% of cancer deaths associated with risk factors that are modifiable [1]. Significant variation in the reducible burden of cancer exists by geographic location. This has led epidemiologists, geographers, and statisticians to use maps and spatial analysis methods to explore and analyze the disease burden in specific geographic locations. By presenting the spatial and temporal aspects of health, health atlases support exploratory analysis, hypothesis generation, and decision-making. In the past, maps of health statistics have offered etiological evidence that led to the identification of explicit disease risk factors. Recently, atlases have also focused upon etiology and prevention of cancer [2-5].

While printed atlases have led to important insights, they require choices about data depiction, classification, etc. to be fixed by the atlas designer and they do not support easy exploration of patterns within or among data sets. Geographic Information Systems (GIS) address some of the limits of paper atlases by offering the potential for users to make their own choices about what data to view and how to analyze them to understand patterns within and across data sets [6,7]. But traditional GIS is expensive, requires substantial training to learn how to use, has limited flexibility for exploring data, and when used for web-based information dissemination gives up many of its advantages (often reverting to static image-based maps or otherwise inflexible maps). Geovisualization research, with its focus on highly interactive interfaces to geographic information, has developed many methods to support more flexible data exploration than those found in typical GIS [8-10]. But like GIS, most geovisualization tools were developed for experts, require substantial training to use, and are designed as desktop rather than web applications.

Despite the fact that GIS, geovisualization, and atlases have provided some success stories, evidence-based best practice methods to analyze and interpret the geographic variations in cancer data have not been developed. Best practice methods could assist policy-makers, program directors and epidemiologists as they implement Comprehensive Cancer Control, a coordinated public health response to reduce cancer risk, improve cancer detection and treatments, and increase access to health and social services.

A key goal in the research presented here is to develop a strategy for map-based information dissemination about cancer incidence that integrates successful ideas and methods from printed atlases, GIS, and geovisualization. Specifically, the goal has been a hybrid GIS-based, highly interactive web atlas that includes flexible links to a database (to support easy addition of new data), information retrieval about specific places, access to tabular data, ability to modify map characteristics, and support for geographical and statistical overviews of data as well as "drill-down" to specific details.

In this paper we introduce the Pennsylvania Cancer Atlas (PA-CA), a model atlas designed as a reference to support cancer control efforts. Developed as part of a cooperative agreement between the U.S. Centers for Disease Control and Prevention and the Association of American Medical Colleges, the PA-CA makes available interactive maps, graphs, and tables depicting colorectal and prostate cancer incidence by county for Pennsylvania. Users of this Atlas are able to quickly explore relevant data as they plan, implement and evaluate cancer control initiatives.

The PA-CA was developed using an evidence-based, systematic, user-centered design process in which input from a range of individuals was solicited throughout the stages of design, implementation, and system refinement. A key part of our user-centered design methodology involved use of a web application to support distributed user input and discussions. This application, called *e-Delphi*, was designed originally to facilitate group-based Delphi exercises via the web (Delphi is a method for structuring a group discussion about a complex problem, usually with a goal of creating a forecast). Instead of conducting traditional Delphi exercises, we applied this tool in new ways to support both individual survey responses directed to specific utility and usability questions about the Atlas as well as to support distributed, anonymous, and asynchronous focus group discussions.

In the section below we present the background and principles on which the PA-CA is constructed and a description of its different components and interactive behaviors. In the Methods Section we present our evaluation methodology utilizing the e-Delphi tool to support the user-centered design process to improve the Atlas. The Results Section reports the results of our four evaluations, using as participants: graduate students, experts of cartography and information visualization, Atlas project advisors, and public health professionals who work for the state of Pennsylvania. Finally the Discussion Section tells us how the user studies have been incorporated in development of the Atlas, and the Conclusion Section summarizes the key web atlas design insights achieved.

### The Pennsylvania Cancer Atlas (PA-CA)

The Pennsylvania Cancer Atlas (PA-CA) is a model, GIS-based, web atlas designed to present current and timely cancer data to inform health care research and policy [11]. As a model atlas, the goal of the research has not been to develop and implement a production system, but to develop an adaptable strategy for such a system and to learn about user needs in online atlases and obtain systematic input that informs atlas design and leads to guidelines for dissemination of the concepts as well as the tools.

Research on the use of health atlases has shown that typical atlas users want to be able to read rates from the maps, recognize regional patterns or clusters in the data, and compare patterns between populations by age, sex and race [12]. This has contributed to the evolution of printed health atlases from atlases with single maps for each disease to atlases that present a combination of maps and graphs for each disease, permitting greater exploration of the underlying data. These user goals have also driven design decisions in previous desk-top geovisualization tools [13]. The PA-CA builds on past advances in both print atlases and interactive geovisualization tools targeted toward cancer data analysis and related public health applications and it incorporates complementary advances in web-GIS and in exploratory geovisualization.

Core design goals that underlie the PA-CA include enabling users to easily obtain geographical and statistical overviews of cancer incidence rate data and to obtain specific details about the rates mapped and plotted. An additional goal is to provide an ability to create and inspect multiple maps of multiple cancers or demographic groups for a particular cancer, in order to support effective comparison and pattern recognition. Finally, the PA-CA is intended for a wide range of users with varied expertise in mapping, thus it is important for the Atlas to be much less complex than a desktop GIS intended for use by geographic information technology experts.

**Figure 1:**
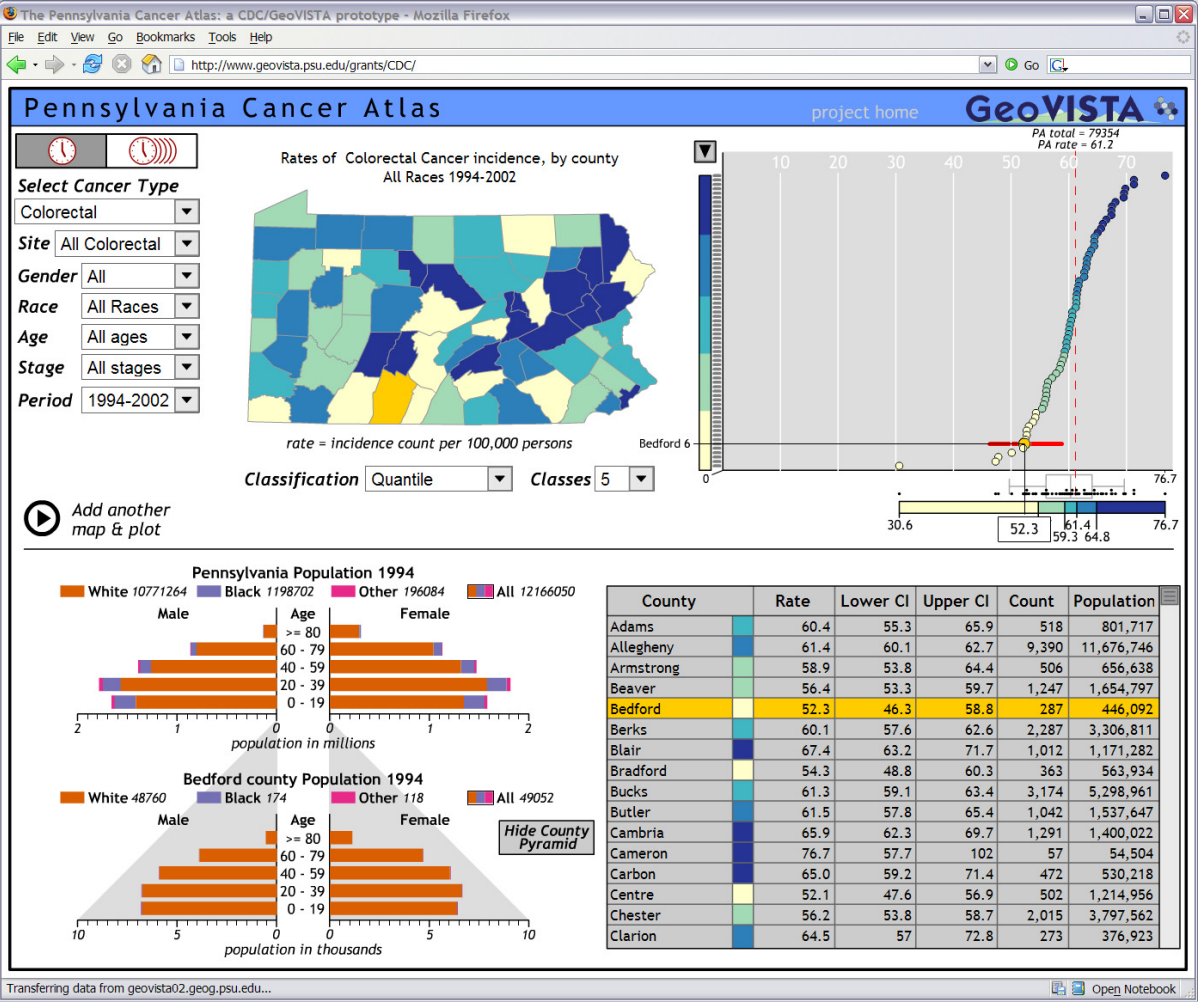
**The Pennsylvania Cancer Atlas, viewable on the web at **

The PA-CA is implemented using a client-server architecture that allows users to request and access cancer incidence data interactively in a web-map client that sends the request through a web server (implemented with GeoServer) to a geospatial database (implemented with PostGreSQL, an open source database, and the open source PostGIS extension [14]). Retrieved data are projected into maps and other data representations using the web mapping client that we constructed using Macromedia Flash client (Figure 1). Details on the architecture of the GIS and its Atlas client are presented elsewhere [15]. Here, we focus on the client web atlas application, our user-centered design process, and general web atlas design guidelines derived.

The PA-CA is currently configured to display colorectal and prostate cancer incidence rates for 1994 through 2002. The primary view is a dynamic choropleth map that depicts age-adjusted rates of incidence for the 67 counties of Pennsylvania. Users can choose the gender, race and age of the population, the site and stage of the cancer, and the time period to be mapped. Users also have the choice of quantile or equal interval data classification methods for assigning data values to classes. Data can be divided into two to seven classes, and classes are colored from low to high mortality incidence using a sequential scheme from ColorBrewer [16]. As a result of the evaluations we report here, we have incorporated a temporal animation feature to animate the maps through three-year rolling averages of incidence rates.

Here we would like to point out the reason for our choice of choropleth maps over other competing models of health data representation such as smoothed maps, which have been argued as being a useful method of representation for health data. The choice is based on input from an advisory committee composed of university researchers and public health professionals at the national, state, and local levels. The overall goal for the PA-CA was to provide a tool that will support policy decision-making and resource management. Since public health is typically organized at a geopolitical unit (e.g., county), public health policy and management decisions that have a geospatial component will have a geopolitically based response [17]. Therefore, choropleth maps that depict each county individually are needed. The fact that none of the advisory committee members or participants in any of the three e-Delphi groups argued for a shift to smoothed maps supports this decision. This is not intended to negate the utility of smoothed maps of disease incidence as they too provide unique information [18].

A dynamic cumulative frequency plot provides a statistical overview of the data set selected; it appears to the right of the map. When a user points the cursor at dots on the plot, the corresponding county's value and overall rank are displayed. In addition, the 95% confidence interval for the rate is represented with a horizontal red line centered on the dot. The plot is linked to the map and table; the selected county is highlighted in all three views (and brushing over features in the other views works in the same way). There is also a line in the frequency plot indicating the Pennsylvania state-wide incidence rate, as well as stating its total count of cases. The legend for the map is included at the bottom of the frequency plot. It indicates the values that define the classification breaks, represents the range of each class with colored bars, and includes a dot plot depicting the specific values and a box plot depicting key characteristics of the value distribution. In the example, Bedford County has an incidence rate for all colorectal cancer (for both genders, all races, all ages, and all stages) over the full time span for which data are available that is well below the median, but that is not an outlier.

The table in the lower right corner of the interface lists all of the counties in Pennsylvania with their associated population and incidence rates, upper and lower confidence intervals and counts. Each county has a color swatch assigned that corresponds to the class it is in on the map. As shown, highlighting is linked between the table and other views.

The lower left corner of the PA-CA interface displays a population pyramid of Pennsylvania with horizontal bars representing males and females in 20 year age intervals. Different colors in the bars represent white, black and "other" populations. The pyramid represents population data for the year or year range selected for the map. By default, the pyramid depicts data for the entire population. Users can focus in on a particular race category by clicking the boxes indicating the color assigned to each category. When a user clicks on a county in the map, a second population pyramid for the county appears below the state pyramid; this pyramid implements the same user controls as the state pyramid. The gray shadow around the county pyramid indicates the number of people from the full state population who reside in that county.

**Figure 2:**
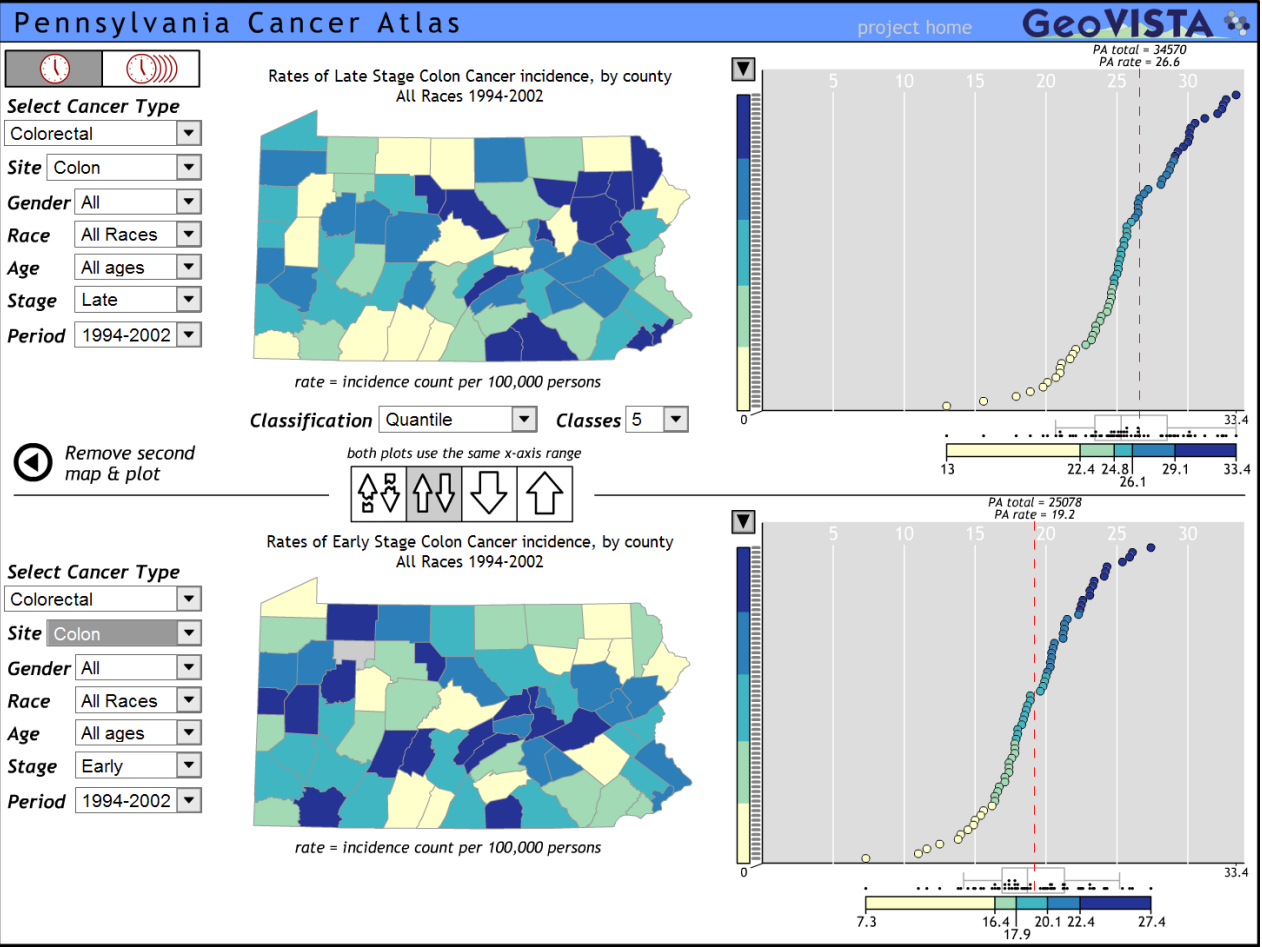
**Comparing geographic and statistical patterns**. For Pennsylvania, the overall state incidence rate for late stage colon cancer (for genders, all races, and all ages aggregated for the period of 1994–2002) is substantially higher than early stage incidence rates but the frequency distribution across the range is similar. The geographic pattern is clearly different; there is a region with late stage diagnosis in the northeast and early stage diagnosis is primarily in central, western, and southern counties. Only Adams, Cameron, and Juniata counties are in the highest rate category for both late and early stage. When explored at more detail, for in-situ, localized, regional, and distant, all three counties are in the highest category for all diagnoses with the exception of Adams and Juniata for localized. For localized rates, Adams is in the second lowest category and Juniata in the middle. For Juniata, the anomaly may be due to the small numbers reported (which are reflected in a large confidence range). For Adams, however, the confidence range is much narrower, indicating that the disproportionately low incidence rate for localize diagnosis is due to something other than chance.

Two other important features of the PA-CA include support for animation through time series data (accessed through the clock-like icons in the upper left) and support for comparing two maps and matched frequency plots. To access the latter, users click the "Add another map and plot" button at the lower left of the main map (figure 2); this replaces the population pyramid and table with a second map and associated cumulative frequency plot. The paired maps and graphs allow users to easily explore similarities and differences between two cancers or two demographic or diagnosis subsets for the same cancer; figure 2 compares late stage with early stage diagnosis for colon cancer incidence rates (over the full times space, for both genders, all races, and all ages).

The components and features presented in this Atlas are designed to promote an understanding of the geographic variability of cancer and the differences in that variability across cancers, race, age, gender, and diagnosis. The interactivity of the Atlas supports this understanding by providing a way to explore the connections between the geographic and statistical attributes of the Pennsylvania cancer data quickly. Viewing two maps and plots at the same time also contributes information by permitting easier comparisons between populations.

## Methods

### User-Centered Design and Utility/Usability Assessment Methodology

As noted previously, the focus of this paper is on an iterative user-centered design process. Iterative user-centered design has been used in determining usability of internet based Geographic Information Systems (GIS) services [19,20], in GIScience[21,22], in Information Visualization [23,24], and in many other domains [25] to improve the usability of software tools so that they can be better suited to the tasks of the end user.

#### Web-based user input

In this paper we adopt a user-centered design approach to incorporate systematically obtained user feedback into each stage of the PA-CA development, thus supporting evidence-based atlas content and design decisions. To iteratively assess and obtain user input toward an effective PA-CA, we use multiple complementary opinion and knowledge elicitation techniques, including focus group discussions and surveys (open and closed ended).

As mentioned previously, we have adapted the GeoVISTA Center's online e-Delphi web application to support distributed input to our user-centered design process [26]. *GeoVISTA e-Delphi *is a web service-based application that was developed as part of an NSF Infrastructure project to develop the Human-Environment Regional Observatory (HERO) Grant No. 9978052. That project was completed in 2007. The HERO project team supported several applications of *GeoVISTA e-Delphi *by external groups during the period of that grant. Plans are being developed to re-engineer and extend *GeoVISTA e-Delphi *and to then provide access for external users.

**Figure 3:**
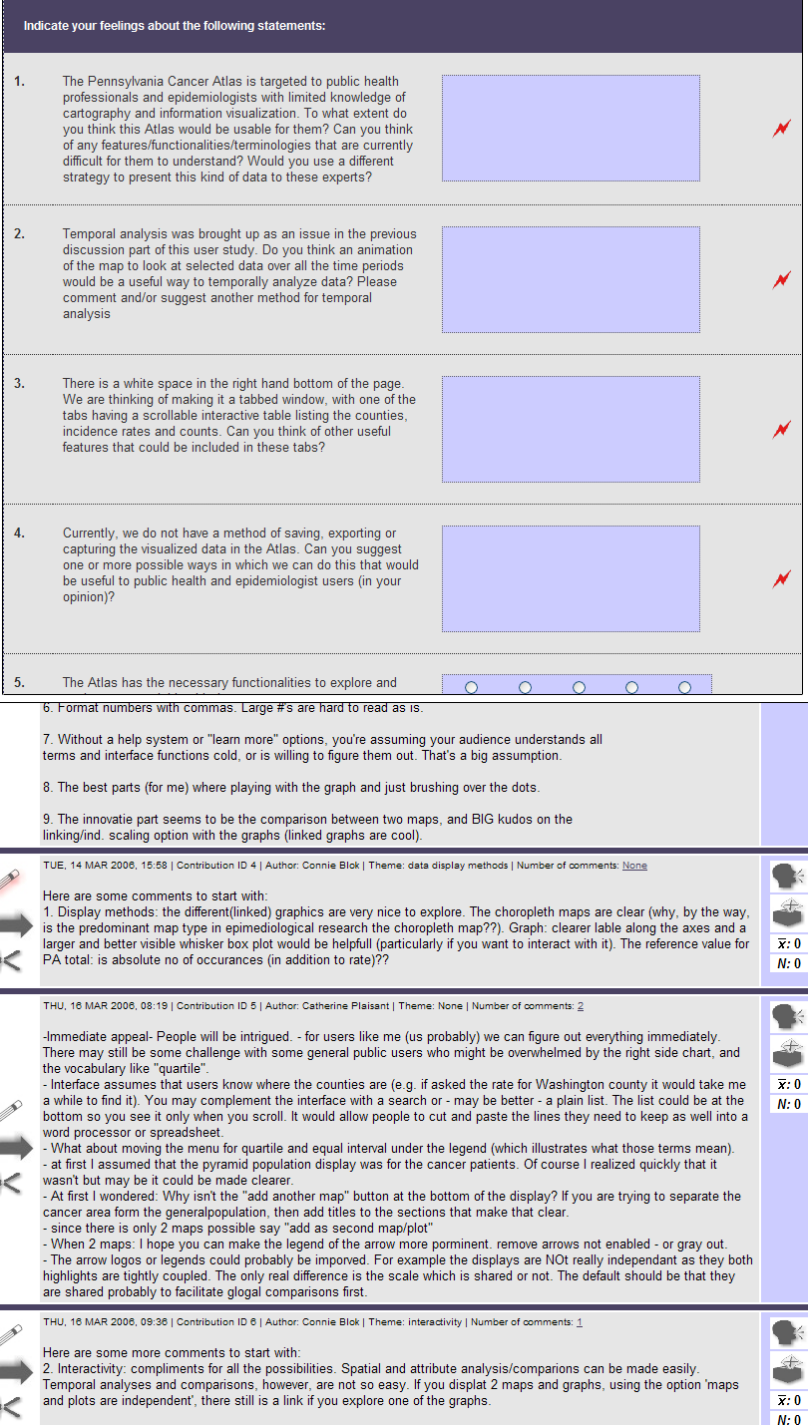
Survey and discussion examples from the e-Delphi interface.

The e-Delphi application was designed to support a Delphi process (i.e., a process intended to develop consensus or more simply to identify patterns of belief, areas of agreement or divergence of opinions). The Delphi method is described in detail by Dalkey [27] and Linstone and Turnoff [28]. The GeoVISTA Center's e-Delphi application was previously used to carry out a Delphi exercise focused on vulnerability science by MacEachren et al[29]. We used the e-Delphi application here with our user-centered design process since it includes support for surveys, free responses, rating, voting, threaded discussions, and moderated discussions (Figure 3).

Our goal was to obtain structured input on the utility and usability of the PA-CA. Thus, we used the available suite of online tools in the e-Delphi application to support several kinds of user input at each of several stages of the Atlas's development. These tools support structured input from both single individuals as well as from groups. Advantages of web-based tools to support these goals (over in-person or mail surveys and in-person group discussion) include: (a) participants can be at any location, eliminating possible travel and cost restrictions for participants, (b) data collection is asynchronous, so participants can provide input at times convenient to them, (c) user input is collected digitally, eliminating transcription errors, and (d) users can remain anonymous during group assessments, reducing inhibitions to comment openly.

#### Assessment Groups

**Figure 4:**
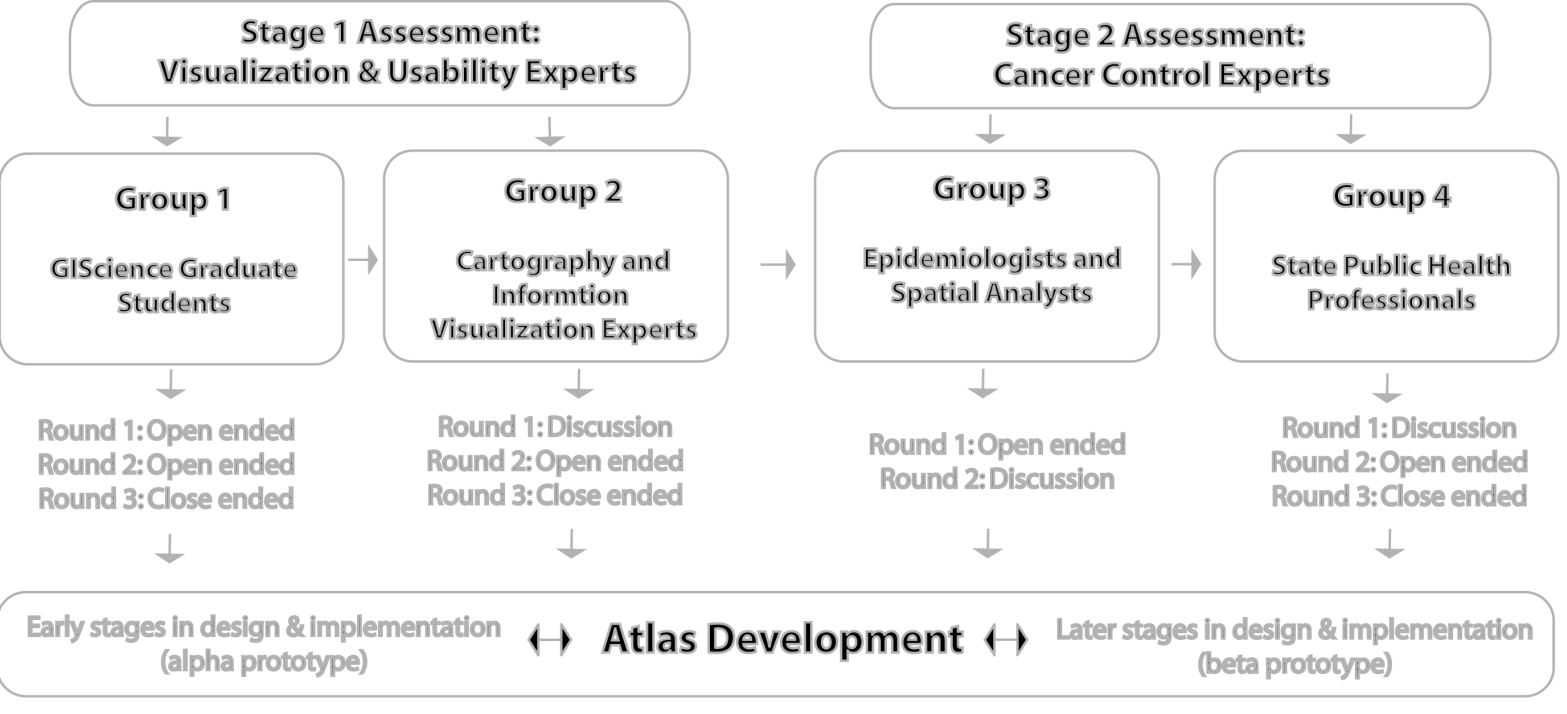
Case studies to evaluate the Atlas development process.

Expert evaluation is a common usability technique within a process of user-centered design [30] and is one we used to support design and implementation of the PA-CA. We carried out four rounds of assessment, feedback, and evaluation with different groups of experts (Figure 4). Results from each round have shaped the design of the Atlas to help improve its utility and usability, and provided input toward development of web-based health atlases, generally.

As illustrated in figure 4, two primary stages of assessment were done. For each, we recruited two categories of participant groups. The stage 1 assessment was done with visualization and usability experts. Stage 2 focused on input from individuals representing the target audiences.

Stage 1 assessments were to evaluate early PA-CA design (what could be considered an *alpha *prototype). We recruited information scientists and designers, many with expertise in geospatial information representation and analysis. Group 1 included GIScience graduate students (at the author's institution) with expertise in geographic data, information systems, and mapping. Most knew of the Atlas project but were not involved in its design. Group 2 included cartography and information visualization experts (faculty in GIScience or information science with expertise in geovisualization or information visualization), all of whom also have expertise in the study of human interaction with map-based displays. None were familiar with the Atlas or its design prior to the study.

In Stage 2, we obtained input from cancer control experts, to evaluate further design and implementation of the PA-CA (the *beta *prototype). We recruited individuals with expertise in the domain which the Atlas is targeted (comprehensive cancer control). Group 3 consisted of epidemiologists and spatial analysts who are members of the PA-CA advisory panel. These individuals represent a portion of the Atlas target audience and had prior knowledge of the design goals and early implementation. We expected them to be both informed critics in terms of achieving design goals while also being tolerant of implementation flaws at an intermediate point in the user-centered design process (thus, they would not be unduly distracted by or focused on features that were not yet implemented or not yet fully functional). Group 4 consisted of state public health officials, representing a key target audience for the PA-CA; they had no prior knowledge of the Atlas or its development.

We identified four categories of information as important to receive feedback on for the iterative development of the Atlas: (1) application design: focused on the Atlas as a whole; (2) tool design: focused on individual components such as map, graph, controls and their functionality; (3) data issues: focused on what the Atlas represents or should represent; and (4) analytical capabilities: focused on support for particular applications of the Atlas. These four categories provided the framework for our prompts and questions to participants as well as our discussion of results for all the user groups. With each group of participants, a combination of survey methods (both open-ended and closed-ended questionnaires) and discussion methods (distributed focus groups) were used to facilitate a broad range of feedback. These diverse data helped us triangulate results. The following sections describe in detail the process and results from each of the two assessment stages that we have completed.

## Results

### Stage 1 Assessment: Visualization & Usability Experts

This section reports on the two rounds of assessment and Atlas enhancement that rely on input from experts in geo/information sciences. Emphasis here is on assessing and improving the cartographic and interface design of the PA-CA as an online interface to geospatial information. Participants in this stage have little or no expertise in the application domain of comprehensive cancer control but do have substantial expertise in geospatial and related information systems, in cartographic and/or information design, and in human-computer interaction. The end goals of this stage in our user-centered design process include well designed maps and graphics, logical dynamic connections among views in the display, logical methods of interaction, and a generally learnable and usable interface.

#### Users and Procedures

The first round of our distributed evaluations for the PA-CA was conducted with a group of seven graduate students who are involved in GIScience research at the Penn State GeoVISTA Center. These students have a wide range of interests, and are all well versed in the application and design of GISystems, mapping, and related technologies.

We asked Group 1 to respond to three sets of questions. The first and second sets contained open ended questions [31,32]. These focused on understanding three major goals: (a) comparing the PA-CA with other similar techniques and applications; (b) assessing the general usability and design of the PA-CA and (c) assessing the match between design and functionality and the target use and users of the Atlas. The third question set contained closed ended questions answered on a five point scale of "Strongly agree" to "Strongly disagree". The latter questions are based on the System Usability Scale method proposed by Brooke [33]. Each set of questions are listed in Table 1.

**Table 1:** Prompts and Questions for User Group 1


**Stage 1 Assessment: User group 1**	**List of Questions**

Round 1 (open ended questions)	1. How have you analyzed geographic information on cancer before?
	2. What is your experience viewing data in choropleth maps, frequency plots or population pyramids? What are your opinions on these methods to display data?
	3. Based on your previous experience viewing maps and working in interactive environments, how logical is the presentation of the cancer data?
	4. Comment on the interactivity of the three information displays. Are they difficult or easy to interpret? Are the connections between them clear?
	5. What are some operations, or ways to manipulate the data, that you found missing and would have liked to have in the atlas?
	6. What additional information or features should be provided in the extra space we have available in the lower right corner of the display? Why?
	7. What features and/or operations within the atlas do you like? Why?
	8. What features and/or operations within the atlas do you feel need improvement? Why?

Round 2 (open ended questions)	1. In your opinion what groups of people could/would use this kind of web-based atlas?
	2. What do you think those groups of people would use this atlas for?
	3. What would you use this atlas for? How often?
	4. What would state agencies need in an web-based atlas to make accurate and timely interpretations of cancer registry data?
	5. How could this atlas assist in planning and evaluating initiatives to reduce cancer morbidity? And cancer mortality?
	6. Do you think this atlas could assist state policy makers in decision making as they implement cancer control measures? Why?

Round 3 (closed ended survey style questions)	Responses to these questions include a five point categorical scale which ranges from "Strongly agree" to "Strongly disagree".
	1. I think that I would like to use this Atlas frequently.
	2. I found the Atlas unnecessarily complex.
	3. I thought the Atlas was easy to use.
	4. I think that I would need the support of a technical person to be able to use the Atlas.
	5. I think that I would need detailed help and tutorials to be able to use the Atlas.
	6. I found the various functions in this Atlas were well integrated.
	7. I thought there was too much inconsistency in this Atlas.
	8. I think that most people would learn to use this Atlas very quickly.
	9. I found the Atlas very cumbersome to use
	10. I felt very confident using the Atlas.
	11. I needed to learn a lot of things before I could get going with this Atlas.

Stage 1, Group 2 included four participants – two information visualization experts (both active academic researchers with some work on map-based visualization environments and on interface usability) and two cartographic experts (both active academic research cartographers who have developed geovisualization methods and conducted usability studies). Three rounds with varied form were used to elicit information from this group; the first was a moderated discussion round, the second consisted of open ended questions, and the third was a closed-ended survey round. In the discussion round a moderator began the discussion by specifying three initial prompts related to various aspect of the Atlas development (Table 2). After participants responded to the three initial prompts, the moderator instructed them to comment on contributions made by other participants. The moderator sent regular prompts to the participants to check back and contribute regularly to the discussion; the discussion was carried out over 4 days. The asynchronous, distributed discussion format provided a method to elicit information on broad issues and problems that the users identified as relevant for the design and functionality of the various Atlas components.

**Table 2:** Prompts and Questions for User Group 2


**Stage 1 Assessment: User group 2**	**Discussion prompts/Questions**

Round 1 (Discussion round)	Initial Prompt: Explore the Pennsylvania Cancer Atlas at: and comment on the following:
	1. The data display methods used
	2. The interactivity between the various information displays, and
	3. The aesthetic design and usability of the interface
	Please make separate contributions for each of the themes listed above and assign the appropriate theme to each contribution.

Round 2 (Open ended questions)	1. The Pennsylvania Cancer Atlas is targeted to public health professionals and epidemiologists with limited knowledge of cartography and information visualization. To what extent do you think this Atlas would be usable for them? Can you think of any features/functionalities/terminologies that are currently difficult for them to understand? Would you use a different strategy to present this kind of data to these experts?
	2. Temporal analysis was brought up as an issue in the previous discussion part of this user study. Do you think an animation of the map to look at selected data over all the time periods would be a useful way to temporally analyze data? Please comment and/or suggest another method for temporal analysis
	3. There is a white space in the right hand bottom of the page. We are thinking of making it a tabbed window, with one of the tabs having a scrollable interactive table listing the counties, incidence rates and counts. Can you think of other useful features that could be included in these tabs?
	4. Currently, we do not have a method of saving, exporting or capturing the visualized data in the Atlas. Can you suggest one or more possible ways in which we can do this that would be useful to public health and epidemiologist users (in your opinion)?

Round 3 (Closed ended, survey style questions)	Responses to these questions include a five point categorical scale which ranges from "Strongly agree" to "Strongly disagree".
	1. The Atlas has all the necessary functionalities to explore and analyze geospatial health data.
	2. The Atlas is a novel approach to access and explore geospatial health data.
	3. The brushing and linking techniques between the map, the frequency plot and the population pyramid is very useful to explore geospatial data.
	4. Aesthetically, the Atlas violates all cartographic principles and design issues.
	5. The Atlas will be very useful for the target audience who are not cartography and visualization experts.

Following the iterative, asynchronous discussion we generated a set of open-ended questions designed to follow up on specific issues raised in the group discussion, to address our overall goal of assessing the PA-CA, and to provide input to future adaptation of the PA-CA approach to related application domains (e.g., atlases for other states, for the U.S. as a whole, etc.). We followed this step with a closed-ended survey that participants completed individually. The goal of the survey was to gauge the overall reaction of this group to the Atlas.

#### Results

The following sections describe the feedback we received from our *Stage 1 Assessment*, combining results from group 1 and group 2. As mentioned in the methods section, we have grouped our results into four sub-categories: application design, tool design, data issues, and analytical capabilities. In the last section of the results we also discuss the modifications done to the PA-CA based on user feedback from stage 1.

#### Application Design

In this section we discuss responses from Group 1 and Group 2 related to the design of the application as a whole. For Group 1, reaction to the layout and presentation of each tool in the PA-CA was largely positive. Users appreciated the simplicity in form and function of the PA-CA, and they considered it to be quite similar to a well-designed paper atlas in terms of presenting organized and useful information. Participants in Group 2 pointed out several consistency issues regarding sizes and styles of fonts, labels and scrollbars. The two cartographers in Group 2 felt that the Atlas followed common cartographic design principles, while the other two were unsure. No specific design issues were identified by the information visualization experts about the PA-CA's cartographic design. These observations lead us to believe that the Atlas can be considered cartographically sound.

Participants in both groups were specifically asked to consider the interactive behavior of the PA-CA. The graduate students in Group 1 had positive things to say about the linked-brushing feature, claiming that it aided quick interpretation by connecting data across views. Some of these users had trouble changing the population pyramid between county and statewide views due to a bug we had not discovered previously (this bug was then fixed prior to the assessment round with Group 2). Group 2 participants were also positive about the interactivity of the PA-CA. They said that the PA-CA provided an "... interesting and novel approach to exploring health data." and the links between the interface components were especially useful for this purpose. Group 2 participants found that the interaction between the population pyramid and the map was not very intuitive.

One weakness of the PA-CA implementation was a lack of application-wide help features and this omission was frequently mentioned by participants in both groups. In Group 1, users stated that the PA-CA's primary weakness was its lack of tool tips or other help features to explain classification methods and related features that are not self-explanatory for novices. Group 2 participants agreed and suggested that providing detailed tutorials would be useful. They concurred with users in Group 1 that it might be difficult for non cartography-information visualization experts to understand the interface without training. To address this, they suggested we incorporate a help system in the PA-CA.

#### Tool Design

In Group 1, each participant indicated prior experience using choropleth maps and had seen or heard of frequency plots and population pyramids. Opinions regarding the suitability of these methods to an online cancer atlas were largely positive – most users felt they would be interpreted intuitively even by non-experts. Some mentioned that the frequency plot may be confused with a more traditional scatter plot. Participants in Group 2 suggested that the cumulative frequency plot would be more understandable with labels added to its axes.

With respect to the choropleth map, the most common request from participants in Group 1 was to allow the selection of multiple counties to make comparisons between groups of places. Users suggested we follow common interface conventions to do this, by using bounding boxes and/or shift-clicking. Two users in Group 1 also specifically mentioned a desire to manually change the class breaks and color schemes to modify the appearance of the PA-CA. This suggestion, however, seems to contradict the reaction from the same group that even the simple, standard classification methods implemented may not be understandable without a tutorial or help tools. Participants in Group 2 suggested we change mouse-over actions to display labels transparently in order to see the map below. Group 2 also suggested that we shift the position of the map legend to a space directly under the map to avoid confusion. These participants apparently did not notice that the current legend is dynamically linked to the position of points plotted on the cumulative frequency plot, with the width of zones in the legend scaled to match the data ranges included. That these knowledgeable participants made this suggestion reinforces the suggestion to provide integrated help tools to supplement the Atlas.

The PA-CA version we evaluated with the first two groups had a blank quadrant in the lower-right of the display. At that stage in development, we had not yet made a decision about what information to provide there, so we asked users in the first two groups to provide suggestions about the best use of this space. The general consensus across both groups was that this space should be used to provide metadata to describe in detail how rates are gathered and what they mean. This could also include general information about each cancer type, who it generally impacts, and common treatment methods. Some users suggested that this empty space would also be a good place to put help files.

While not mentioned in Group 1, participants in Group 2 focused attention on the lack of a temporal component to the Atlas. As tested, the PA-CA allowed some temporal exploration when two maps were displayed together showing different time periods. Group 2 participants suggested we focus attention on incorporating tools designed specifically for temporal analysis. Potential solutions suggested included a trend analysis plot, time line tool, map animation tools, and a time slider bar for easy user interaction.

#### Data Issues

In Group 1, two participants responded that they had prior experience working with geographic data on cancer. The rest stated no familiarity with the topic. We did not specifically ask Group 2 participants about this because we were familiar with their published research and knew that three of the four had worked on topics related to various types of medical data in the past.

Participants in Group 1 suggested that end-users may want to change the default map aggregation units to zip code areas, tracts, and congressional districts. While it is obvious why these options would be useful, serving geographic data at resolutions finer than counties is impractical due to confidentiality concerns and congressional districts often split counties, requiring completely separate processing of the original individual data to aggregate to these districts (something impractical for this model atlas, but feasible for a final product). Participants in Group 2 again pointed out the need for access to metadata and specific variable descriptions. In Group 2, participants indicated the need to supplement the interface with a list, table, or similar methods to provide a better overview of the available data. Participants suggested we provide a table from which users could copy and paste attribute values into an external spreadsheet. When we asked more generally how the users envisioned data capture or export from the PA-CA interface, Group 2 participants suggested screen captures, PDF, and power point exports as useful output formats. These responses indicate a desire for both map/graph export and data export. For data, since the web-mapping client is linked directly to a database, it would be possible to provide an option to generate data files for download; but including this option would only be possible in cases where the agency providing the information made a policy decision to distribute data in addition to providing interactive maps.

#### Analytical Capabilities

In Group 1 we asked participants to describe potential applications for the PA-CA. Their ideas were primarily related to public health analysis and decision making. A few felt that the Atlas could be used to teach map literacy and cartographic design. Group 1 participants also described scenarios in which analysts would use the PA-CA to identify areas where health education and screening efforts could be targeted, or to monitor the effects of existing education and screening campaigns over time. Because the PA-CA was seen as generally easy to use, Group 1 participants indicated that decision makers might also use the PA-CA to create graphical reports for information dissemination. The need to support display capture in a format that enables integration into reports echoes ideas that our advisory committee had offered earlier (prior to the initial prototype) and is similar to suggestions made by participants in Group 2 when they discussed data export features. Some Group 1 participants also suggested that the PA-CA could be used to evaluate claims about particular risk factors (perhaps environmental or social) in certain areas. The idea that a map can be used to "evaluate claims" is, of course flawed. Maps are good at prompting users to notice potential relationships that should then be followed up on through accepted statistical analysis methods. Agencies adopting web-mapping technologies should include integrated tutorials that help users understand the resulting maps and how to use them appropriately.

Participants in Group 2 generally agreed (one participant was not sure) that the PA-CA has the tools and features necessary to explore and analyze geospatial health data. Since none were experts in the domain, they did not suggest specifically how the PA-CA might be used for comprehensive cancer control. One participant in this round suggested that this was something we should evaluate with the health analysts we had in mind – this is precisely what we had planned for Groups 3 and 4.

#### Atlas Updates Based on Stage 1 Assessment

As indicated above, several important suggestions were made and issues were raised by the Stage 1 participants concerning design and functionality of the PA-CA. As a result, several small adjustments were made to the Atlas and three major features were added. The first two address suggestions about the need to add more information to allow users to understand patterns they see and to access information that the patterns prompt them to be interested in (essentially support for the classic information visualization idea of supporting overview, zoom and filter, plus details on demand). The third addresses the call for a mechanism to explore the temporal component of information provided. Specifically, the three major additions following Stage 1 of assessment were:

1. A scrollable table was added to the lower right quadrant of the Atlas web-client. The table is one of several features suggested. Thus an implementation strategy was developed that allows the table to be one of multiple tabbed information access components available (one at a time) in the lower right position of the display.

2. The population pyramid was extended to show details of counties and sub-divisions based on different racial categories.

3. An animation feature was added to the Atlas to show temporal changes for selected variables over time.

Beyond these three major additions, two key additional changes were also made:

1. The population pyramid bug which prevented it from resetting properly was fixed.

2. Font styles, types and sizes were made uniform; scroll bar styles were made uniform; numbers were formatted with appropriate commas; units and titles for maps and graphs were placed more logically.

There were several other important suggestions made by the users at this stage. Users requested an integrated help system, and they also suggested an interactive glossary of definitions. We have made plans to update the PA-CA with these changes, but they were impractical to implement before State 2 of assessment. These and many other suggestions for improving the Atlas are summarized in the discussion section where we discuss the overall set of suggestions for improving the PA-CA generated by both stages of assessment.

### Stage 2 Assessment: Cancer Control Experts

This section reports on the two rounds of assessment and Atlas enhancement that rely on input from cancer control experts, epidemiologists, and spatial analysts. Emphasis here is on assessing and improving the utility and usability of the Atlas for the target audience. Participants in this stage generally have limited expertise in visualization and usability assessment, but do have expertise in the application domain of comprehensive cancer control. The end goal of this stage of the user-centered design process followed in this research and development project is an interactive Atlas that is intuitive and usable as well as useful for the target community of interest.

#### Users and Procedures for Stage 2

This stage of our assessment uses two primary user groups (refer to Figure 4 for an overview of our assessment stages and user groups). Group 3 included members of the PA-CA advisory committee, thus these participants had specific prior knowledge of the goals against which the Atlas was being judged since they helped to set those goals. Group 4 represented the broader community of public health professionals who are typical target users; they had no knowledge about the Atlas prior to being asked to use and assess it.

Group 3 contained seven participants; they included epidemiologists, health researchers and spatial analysts. This group was not only familiar with the motivation behind the Atlas but with earlier stages in Atlas design. Thus, they were in a good position to discuss design trade-offs in the context of project goals. Group members each have several years of experience in research on or policy and outreach about cancer and public health more generally. Most also have some familiarity with spatial analysis of health related data or at a minimum the mapping of these data.

Since we had worked actively with participants in Group 3 from the beginning of the project, they were asked to participate in only two rounds of input (in contrast to the three used with the other groups): an open ended survey and a structured and moderated discussion (Table 3). There are several noteworthy characteristics of this group. Since this group had met in person, at two Atlas advisory meetings, we decided to start with the survey round in order to capture individual input before ideas were further influenced by each other's views. The survey round had eight questions, and after the round was over a summary of the answers was made available for the next round of discussion. The survey round was followed by a structured, moderated discussion similar to the one used in our previous rounds with Groups 1 and 2. In the discussion round, participants were asked initially to comment on the summary statements for each of the questions. They were also asked to check back later and respond to other participant's comments. Therefore, the second round had initial comments on the main issues as well as some threaded discussion.

**Table 3:** Prompts and Questions for User Group 3


**Stage 2 Assessment: User Group 3**	**Questions/Prompts**

Round 1 (Open ended questions)	1. What use/uses do you see as the primary ones for this Atlas?
	2. Who within public health would be the primary user of the Atlas? Of the results?
	3. What additional data should be included in the Atlas?
	4. Do you think the links between the four components (map, frequency plot, population pyramid, table) are intuitive and useful? Please explain any specific links that you feel are particularly intuitive/un-intuitive and/or useful/un-useful.
	5. Can you think of other data display methods than the ones you see in the Atlas that are useful from the point of view of cancer data analysis?
	6. We are planning to have a tabbed window at the lower right portion of the interface. We already have a scrollable interactive table in one of the tabs. What other information/tools would you like to see incorporated there?
	7. What is your opinion about the temporal animation feature? Do you think this is a good way to explore temporal data? Are there any alternatives you feel we should consider and/or ways to make the current animation feature more useful?
	8. Are there any features, functionalities, terminologies, etc. which might not be understandable to the target audience? If yes, can you discuss some ways to overcome the problems?

Round 2 (Discussion)	In this section the results of the previous round was summarized and put on a website. The users were given a link to this site before this discussion round begun. They were asked to comment on other people's responses as seen from the summary or raise issues which they thought were important regarding the Atlas.

Group 4 included seven public health professionals who represent our target end-users. These professionals work in state health departments and have substantial experience working with and analyzing cancer data, although they are not especially versed in analyzing the geographic variance of these data. None had prior familiarity with the PA-CA.

Group 4 was asked to participate in three rounds. The first was a discussion round. The group was asked to explore the PA-CA; they were provided with three initial prompts to begin discussion on three broad issues (Table 4). The purpose of this round was to stimulate discussion on data display methods, interactivity of the displays, and data issues within the Atlas. Following this discussion, they were asked to respond to open-ended questions, then to a closed-end survey.

**Table 4:** Prompts and Questions for User Group 4


**Stage 2 Assessment: User Group 4**	**Questions/Prompts**

Round 1 (Discussion)	Initial Prompt:
	Explore the Pennsylvania Cancer Atlas at and add contributions to specifically comment on the following:
	1. The data display methods used and their ability to analyze/explore cancer and health information
	2. The interactivity between the various information displays and the ease of understanding and using them
	3. The kinds of data that has been used in the Atlas and their usefulness for public health professionals. Additional data that could be useful for cancer data exploration and analysis.

Round 2: (Open ended questions)	1. List representative tasks that you feel the Atlas is well suited to accomplish.
	2. Should additional data should be included in the Atlas? If so, what data?
	3. Is the Atlas (in its current state) usable for your data exploration needs? Please explain your response, briefly.
	4. Is it usable for your data analysis needs? Explain.
	5. Do you think you can use this Atlas for presentation of your data? If yes please explain how?
	6. Are the links (dynamic connections) between the four components (map, frequency plot, population pyramid, table) intuitive for you? Please explain any specific links that you feel are particularly intuitive/un-intuitive and/or useful/un-useful.
	7. Please suggest additional data display methods that would be useful.
	8. We are planning to add a tabbed window in the lower right portion of the interface (where we currently have the scrollable table). What kinds of other information or tools would you be useful?
	9. Please provide your opinion about the temporal animation feature. Specifically, do you think this is a good way to explore spatial-temporal data? Please suggest any ways to make the current animation feature more useful.
	10. Are there any features, functionalities, or terminologies which are difficult to understand? If yes, please identify them and suggest ways to overcome the deficiency.

Round 3: (closed ended survey style questions)	Please rate your overall impression of the Atlas on the following five point scale (Strongly Agree to Strongly disagree):
	1. The Atlas is quite easy to use
	2. The Atlas requires detailed help and/or tutorials to be usable
	3. Please rate the overall temporal capabilities of the Atlas on the following scale:
	4. The various display methods in this Atlas are well integrated
	5. In your opinion, the Atlas has all the necessary functionalities to explore, analyze and present geospatial health data.

#### Results

The following sections describe the feedback we received from the Stage 2 Assessment combining results from Groups 3 and 4; as above, results are grouped into four categories: application design, tool design, data issues, and analytical capabilities. We also discuss updates to the Atlas implemented since these two group assessments as well as those that are impractical to implement in this prototype, but that would be important to consider in a production version of the Atlas.

#### Application Design

Feedback on the overall utility and usability of the Atlas was positive from all seven of the participants in Group 3. One participant mentioned that the purpose of the tool should be clarified and another suggested that the legibility of the box plot should be improved. Six of the seven participants in Group 4 thought the Atlas was generally useful. The seventh participant rated it as fairly useful. All participants from Group 3 agreed that the Atlas was relatively easy to use, though five thought that tutorials or other help features would be helpful. In Group 4, five out of seven respondents stated that the Atlas was easy to use, while one respondent strongly disagreed and the other had no opinion. We examined the other responses of the person who strongly disagreed and found that this person had trouble getting the Atlas to display properly on their computer.

All seven participants in Group 3 stated that the Atlas' primary users would include state and local health authorities or cancer program planners. Two participants thought it could be an important tool for health advocates as well. Two others suggested it may be of interest to researchers and students, and one participant suggested a potential use for legislators and hospital officials. One participant in this group held the view that the Atlas would not be used by the public, nor was it useful for the "higher-ups". They did not define this group of "higher-ups". The target end users in Group 4, (state health professionals), generated mixed responses on questions about the uses of the Atlas for their own work. Two of the six participants responded that the Atlas would be useful to them; three stated it could be of use to them if data and/or features were added, namely: confidence intervals, mortality figures and a way to print or export the maps; and one person felt that the Atlas was too difficult to use.

All participants in Group 3 indicated that the basic linked interactions in the Atlas were generally useful and intuitive. However, two participants pointed out that the links between the population pyramid and the other data displays were not apparent. One of them stated that it was not clear that the user could select a county in the map to get a county-specific population pyramid, and the other did not notice any links between the pyramid and the other displays at all. Two participants from this group suggested that additional explanations or tutorials would be useful for people not familiar with linked displays. In Group 4, most participants found the linked interactions useful and intuitive, with the exception of one participant who stated that they were not intuitive but, "... easy enough to figure out after a few tries."

Users in both Group 3 and Group 4 suggested adding help files or tutorials to assist users through the different features of the Atlas or to explain terminology. In Group 3, participants brought up the idea of tutorials in reference to certain parts of the Atlas; specifically, to demonstrate the function and features in the population pyramid (suggested by three of the seven participants) and the temporal animation (suggested by two participants). Two participants recommended tutorials to illustrate the dynamic linking between the displays. Another suggestion from this group was to provide a legend of definitions or to provide pop-ups to define terms on mouse-over actions. Four of the seven respondents felt there were some terms that might inhibit proper use or comprehension of the Atlas. These terms were: quantile, align x-axes, in situ, early stage and late stage. Two others felt that the methods behind the terms "rate", "count" and "no data" needed to be explained. In Group 4, three of seven participants agreed that the Atlas needed detailed help or tutorials, while three thought it was fine without help or tutorials, and one participant had no opinion. Explicit suggestions for help features included a simple set of step by step instructions for general use of the Atlas, and tutorials to describe map classification methods and how to use the temporal animation tools.

#### Tool Design

Feedback on the choropleth map was positive overall with a just a few comments on how it could be enhanced. A repeated request from both groups is that there should be a way to export and/or print the map (and other displays). One participant from Group 4 added that there should be a traditional legend with color boxes and data ranges for export purposes to aid interpretation. In Group 3 three people recommended that the map could use the temporal data to show counties with unstable rates or those counties that have increasing or decreasing rates over time. Details about how this information should be calculated or implemented were not provided. One Group 4 participant noted that there should probably be an "unknown" category in the stage dropdown menu. Another recommended signifying the counties that had significantly different rates than the state average (our intention was for the box plot to show distribution characteristics). Another thought that it was confusing to include data for single years, 3-year averages and the whole time period together in the same Atlas. The temporal animation feature generated a number of suggestions and mixed comments from both groups. In Group 3, one participant suggested showing small images of each mapped time period below the main map, and to use them to indicate which map is being shown in the animation as it runs. A third participant from this group stated that the animation is not especially useful in its current state, pointing out that there is no way to select or highlight specific counties to track for persistent high or low rates. This participant also stated that the requirement that the animation be rewound to play it again was not intuitive. In Group 4, two participants remarked that the temporal animation was good. Two other participants found the tool marginally useful, noting that there needed to be a way to track certain counties or highlight counties where there is significant change. Two participants responded to our question about the temporal animation tools with question marks, so it seems that they did not find the feature at all. In both Groups 3 and 4, a number of participants stated that the clock icons used to signify the temporal animation tools were not intuitive.

In Group 3 comments on the frequency plot focused on increasing the size of the histogram and box plot, and there was also a recommendation to display the 95% confidence interval bars permanently to give more meaning to the ranking of the counties, rather than only showing them on mouse-over. Two participants from Group 4 suggested improving the legibility of the legend beneath the plot and labeling the axes of the plot for quicker comprehension. A conventional normal distribution was suggested as a more intuitive option to replace the frequency plot by one participant. Another suggested an option to keep the data range consistent on the x-axis of the plot as various populations or diseases are viewed to enable easy comparisons.

Comments on the population pyramid from Group 3 critiqued the relatively hidden feature of being able to create a county pyramid by selecting a county in the map. One participant recommended the county population pyramids change as the user mouses over the map. Another participant thought that the features of the pyramid might not be easily understood by some users. In Group 4, two participants thought the pyramid was not very useful, even "superfluous," and one participant recommended providing it as a tool that appears only on demand. Another user in Group 4 doubted a general audience would understand its importance in the Atlas. Yet another user thought the pyramid was especially *useful *for cancer control planning.

We asked participants in Group 3 and 4 what additional tools or information they would include in a tabbed window that we plan to place in the lower right corner of the Atlas' display (where the table was when they evaluated the Atlas). Suggestions for the interactive table included adding additional columns of data, data export tools, and column-based sorting capabilities. Three participants (of seven) from Group 3 stated asked for additional depiction of temporal information, two suggested time series plots and the other small multiple maps, both to examine how cancer incidence or mortality is changing over time. Two participants suggested micromap plots, one participant suggested information on risk factors like SES, health insurance or BRFSS data represented in maps or tables, and another suggested scatter plot displays of incidence broken down by population age, sex, and race groups against the overall incidence rate. One person suggested adding the capacity to map potential covariates against incidence or mortality rates. Another suggested providing current information on science based interventions related to the type of cancer being investigated.

#### Data Issues

Participants from both Groups 3 and 4 are, as intended, quite familiar with the domain of cancer control and the data displayed in the PA-CA. Many of the participant's general comments focused on the need for information about how rates are calculated and standardized, and additional variables that would make the Atlas more useful for them.

Participants from Group 3 requested data for other cancer sites as well as a measure for the total cancer burden. A number of participants stated that mortality data would make the Atlas more useful and one person requested early detection and prevention data (i.e. screening exams). Another participant in Group 3 suggested adding specific variables to the table, including: incidence rate standard error, incidence rate upper and lower confidence bounds, rate ratio, rate ratio standard error, and rate ratio upper and lower confidence bounds. A second participant stated confidence intervals should be included and. another recommended showing the rates for different time periods in the table. One participant recommended data on other subsets of the population, and because small numbers might be a problem, to include Poisson probability estimates and/or Bayesian smoothed maps. Three participants from Group 3 made it clear that metadata was needed to describe how rates were standardized, what base population they were calculated from, and how they were temporally aggregated. One participant pointed out that suppression rules used for counties where "no data" is displayed should be explained.

Group 4 participants had similar observations, among them was the suggestion to extend the available data to include additional cancer sites. Three participants from Group 4 also recommended mortality data be present, and one of them suggested confidence intervals, expected values and population totals should be added. Economic, demographic and risk factor data were also suggested as important additions, as were confidence intervals, expected values and county rate goals. Two people suggested showing the data by legislative (congressional and/or voting district) regions, which would be useful for advocacy (although it may be politically sensitive, as one noted). Another person suggested grouping the counties by health district.

#### Analytic Capabilities

To assess the Atlas' analytic capability we asked participants in Groups 3 and 4 what important tasks the PA-CA would be well-suited to perform. Group 3 participants suggested that the PA-CA would be useful for evaluating regional differences in cancer rates, quickly obtaining rates and case counts by county, exploring county rank information, and as a way to disseminate information to the public, which, as one participant noted, could help lessen the number of yearly reports. One participant stated that geovisualization of cancer at the county level could stimulate grassroots interest. Another pointed out that the PA-CA had limited utility for comprehensive cancer control without being able to export the displays and the data. Finally, one participant pointed out that in general the Atlas is a good tool for exploration rather than a tool for hypothesis testing or analytical modeling.

Group 4 participants indicated that the Atlas supports identifying and comparing counties, examining time periods and cancer stages with high, low or unusual rates, and assessing how incidence rates differ across portions of the population. As Group 4 consisted of our target end-users for the PA-CA, we asked them how usable the Atlas was for data exploration, for data analysis, and for presentation of data. Out of seven participants, three thought the Atlas was suited for data exploration in its current state, and one stated that it would be with the ability to export displays and data. Two others expressed that it would be useful for data exploration if it had the additional variables mentioned in the previous section. One participant simply stated that the Atlas was too hard to use (this was the user who had trouble running the PA-CA on their computer). Similarly, for data analysis, three people said that the PA-CA was suitable for data analysis. One noted it would be acceptable only at a macro level, and another stated it would need additional data and the ability to export data and display screens in order to be useful for data analysis. One participant emphasized that it was not suitable for data analysis because there are many specialized ways to analyze data and the PA-CA supports only some of these methods. One of the seven did not answer this question. For presentation purposes, three participants stated it would be useful, three mentioned that it would be useful provided one could export and/or print from the Atlas, and one did not respond.

#### Atlas Updates Based on Stage 1 & 2 Assessments

Many participant suggestions have been or are being implemented in the current working version of the Atlas. Some are beyond the scope of the current model atlas project and others conflict with Atlas goals and or other capabilities. The issues raised and responses to them are described in detail in Appendix 1. A general discussion of these issues follows in the next section.

As mentioned earlier, we are currently implementing a glossary of definitions as a direct result of the comments made by our users in both stage 1 and stage 2 assessments. A step toward a help system has been completed in the form of a narrated movie the PA-CA overall and introducing the key components and how each of them work. This will help users to fully exploit the potential of the Atlas. The glossary of definitions and terminologies will ensure that all the complex terms that might be unfamiliar to the end user are well defined. Therefore this will take care of two major issues identified by all four user groups. Some additional suggestions adopted include: a user control to synchronize the x-axis of cumulative frequency plots in the two-map view and support for sorting in the table view.

## Discussion

Our primary goals for distributed assessment of the PA-CA were to evaluate and improve the usability of the Atlas, evaluate its overall and tool-specific utility for public health professionals, and provide input to design of web maps and atlases for aggregate health data more generally. Here, we review key results of the multi-stage assessment and design process and offer general design guidelines derived from the results.

Overall, the participants perceived the Atlas to be useful, interesting, and usable; the difficulty level was considered to be relatively low. Participants were largely positive about the potential of the Atlas to become a model tool for exploring health data. Based on the reactions, the Centers for Disease Control requested a U.S. version of the Atlas (with state-level data); development of this version was just completed and transferred to CDC for deployment.

The positive overall reaction was balanced by a large number of issues identified by participants in our user-centered design process. The issues suggest possible changes and additions that would enhance the usefulness and usability of the Atlas. They also provide a basis for developing guidelines for web-based map/atlas design and implementation more generally. Across the full development process, the two stages of distributed assessment yielded 66 different issues. We list the full set and our response to each as an appendix to the paper. Since the current implementation is meant to be a model rather than a production application, we did not attempt to adjust the PA-CA prototype to implement all appropriate suggestions that were made. During the user-centered design process, those issues and suggestions that were most critical to enable productive input from the next design round were implemented (if practical). Others are reported above and in the Appendix: Issues and Responses to User Feedback (Additional File 1) to inform design of subsequent web atlases and served as input to the design guidelines provided below.

### Changes and additions to PA-CA during the user-centered design process

Participants in Stage 1 identified a group of issues related to desirable features not in the early prototype; several of these led to key changes. Changes include the addition of three major features and many smaller adjustments. The three major additions prompted by user input were: a time-series animation capability; a scrollable data table dynamically linked to other views; and a county-based population pyramid to complement the state-level pyramid. To create the flexibility for adding a range of supplemental information, a multi-view tabbed window was designed and implemented and the table view was inserted as a tab within this window.

The most important additions prompted by Stage 2 input (from representatives of the target audience) are initial tutorial and help features. The tabbed window provides a mechanism for including some of the help/tutorial material proposed. One suggestion was to add the definition of terms used; a glossary tab has been added for this purpose. As a first step toward tutorials that help users learn about the Atlas and how to use it, we have created a narrated movie explaining Atlas tools and their use; it can also be accessed through the tutorial tab. Many other smaller changes have been made in response to user input, as can be seen by comparing suggestions to the current version of the Atlas.

### Design guidelines for dynamic web-based health maps/atlases

Beyond the direct impact on Atlas implementation, the issues and suggestions generated by participants were core input to the following design guidelines for choropleth-based, web atlases of health data generally. These guidelines synthesize the full set of input from participants and our interpretation of that input.

• Dynamic linking is considered fundamental by information visualization/geovisualization experts and considered to be a potentially valuable enhancement to traditional maps by most of those representing the target audience. To use it effectively, however, requires careful attention to the way connections among views are implemented and provision of clues for novice users to help them realize that (a) dynamic linking is possible and (b) that specific features are linked.

• Help and tutorial features are essential for dynamic web map/atlas tools targeted to users whose expertise is not in geographic information technologies. This support should include easily accessible definitions of key terms and concepts, tool-specific explanations of use, introduction to the overall application and the cross-links among tools, and an explanation of the application that each tool and combination of tools affords. We have produced a sample narrated tutorial that applies a scenario-based approach to introduce both the web Atlas and its potential application.

• As in traditional paper atlases, there is a need for supplementary information (in the form of text, tables, pointers to additional resources) to help users interpret what they see on the maps. An interactive environment has the potential for much more flexible access to such information – and tabbed views are a good strategy for providing easy access to multiple kinds of information. One possible mechanism to provide such information that we are investigating is to embed (in the tab view) RSS feeds that enable continuous updates of such information from PubMed [34].

• Support for exporting the results of what is found when exploring data on maps is essential. At a minimum, screen capture tools are needed that enable export of interesting views as images for presentations. Many users would also like data export capabilities. However, (while technically easy) providing data export is mostly a policy issue related to data confidentiality and other data dissemination policy.

• Users are likely to want flexibility to regroup data by alternative enumeration units (e.g., grouping by Congressional Districts). Providing the flexibility will be constrained by data confidentiality guidelines (as above), but when possible requires a server-client architecture that supports independent back-end data processing and web client display.

• The potential offered by interactive views prompts the expectation that the environment can support flexible information queries, thus it is important to meet these expectations with intuitive visual query tools. For example, different participants suggested the following as desirable: obtaining data by individual and groups of counties quickly, examining regional differences, comparing counties and identifying overall county rank, exploring geographic pattern for time periods or cancer stages with particularly high, low, or otherwise unusual rates.

### Reflections on additional suggestions

Beyond the suggestions and issues raised that prompted direct change in the PA-CA during development and those incorporated in the design guidelines above, there are two that deserve specific attention due to their potential implications.

First, two people suggested adding micromap plots [35] as a view for the Atlas. This representation form has been shown to be quite effective and adopted as a method used on the National Cancer Institute/CDC State Cancer Profiles web site . Thus, there is precedent for using the method and a growing set of potential users familiar with it. However, although micromap plots can be quite useful, much of the same information can be obtained through the use of the map and frequency plot together as implemented in the PA-CA; and the PA-CA format requires about half the screen space to achieve this. One example of a key advantage of micromaps over standard maps when the maps are static is that they make it easy to locate particular data ranges on the map (because each small range is isolated on its own map). This task can be achieved easily with the PA-CA due to its interactivity and linked views. A user can simply hover the cursor over any category (colored area) on the x or y axis of the cumulative frequency plot and see only those counties making up that category in the map and the plot. Plus, by using a click-hold on the category, the color scheme is changed to show the top and bottom half of values in the category with different levels of saturation. This allows users to obtain most of the information found in a micromap within a composite view that makes it easier to notice state-wide patterns.

A very different issue that generated considerable input and many ideas involves how to represent and help users understand change over time. While the animation implemented is a partial solution, a common critique of the temporal animation as implemented is that it lacks the capability to track or highlight counties so that changes in rates and rank over time are easy to determine. We feel that the issues raised and suggestions about understanding change over time are important ones and that additional research and development should focus on how to better support this understanding. Broadly, the key question is how do representation and interactive control choices (separately and through their interactions) enable or impede understanding of change. This question has many subcomponents, some that were made apparent through our user-centered design and assessment process include:

• what are the most effective methods for supporting the interconnected space-time queries of: what, where, and when;

• what are the relative advantages of animation, small multiples, change maps, and other alternatives for understanding components and characteristics of change in both geographic patterns and attribute values;

• specifically, what methods provide the best support for tracking change of an enumeration unit or region over time;

• specifically, what methods best support identification of new spatial clusters or change in position of existing clusters over time?

## Conclusion

The user-centered design approach adopted for the PA-CA, has resulted in a relatively effective working model for similar state or national-level atlases to support cancer surveillance and control or related public health domains. Because the PA-CA is intended to be a model for other state-level cancer atlases, our work with the Atlas has the potential for broad impact. This potential carries with it a responsibility to ensure that we are carefully considering the end-users of these tools. Therefore, as we continue to improve the design of the PA-CA, we will also continue to solicit user feedback through additional evaluation activities.

In general, we are pleased with the feedback we received through distributed evaluation using the modified e-Delphi application. Our work identified important areas for improvement for the PA-CA without requiring time and capital intensive same-time, same-place evaluations. Thus, in addition to insights about design of web maps and atlases to support health data dissemination and understanding, this research has demonstrated the potential of distributed web-based tools to support group input to health geographics tool design.

Finally, beyond the Atlas itself and insights on distributed user input to tool design, the iterative design process, involving input from multiple categories of experts, served as the basis for a set of general design guidelines. These guidelines should be useful for subsequent web-map/atlas design to support public health, regardless of the particular technology used to implement the maps.

## Competing interests

The authors declare that they have no competing interests.

## Authors' contributions

All the authors were involved in the initial design and conception of the study. TB prepared the prompts and questions for the usability testing process, conducted part of the Usability testing and wrote the Results Section for the Group 2, 3 and 4 assessments and part of the Discussion Section. ACR wrote the Methods Section and the Results from Group 1 assessment. He compiled the figures, tables and appendix and contributed to the overall drafting and re-writing of the methods and results section. AG conducted part of the Usability testing process and wrote part of the Background section of the paper. AMM wrote the Discussion and Conclusion Sections of the paper, reviewed and critiqued the paper number of times. EL wrote part of the Background Section, the abstract of the paper and contributed to the overall critique of the paper.

## Supplementary Material

Additional File 1 Appendix. Issues and responses to user feedback Click here for file
