# Supplementary material for: Distributed usability evaluation of the Pennsylvania Cancer Atlas
Source: Int J Health Geogr. 2008 Jul 11;7:36. doi: 10.1186/1476-072X-7-36 (PMC2490686; doi:10.1186/1476-072X-7-36)
Supplement: Additional File 1 — Appendix. Issues and responses to user feedback [file 1476-072X-7-36-S1.doc]

# Appendix

# Issues and Responses to User Feedback

| **User Generated Issues** | **Our Responses and Ideas** |
| --- | --- |
| 1. Some aspects of the UI are hard to read because of small font size. Among them specifically mentioned was the box plot. | After discussion we decided that the current design of the box plot fits the overall design of the Atlas and if you mouse over the feature then the dots are linked with the frequency plot. Changing the size of the box plot would involve changing the size of the frequency plot and there are space constraints in the Atlas. One option for taking a closer look at the box plot would be to right click and zoom in on the box plot for a closer look. |
| 1. Missed/could not easily understand that clicking on a county updates the population pyramid feature. | Making the pyramid update dynamically was thought of (actually suggested in Gr3&4) as an option. However this may make the interface too busy and understanding the different interactions would become much more difficult for an individual to process. Instead we decided to put a label under the population pyramid which explicitly says to click on the counties to update the pyramid below. Outlining this in the help system would make the users understand this feature better. |
| 1. Add another map legend under the map in addition to the one provided with the frequency plot. | We concluded that having a traditional legend below the map would be a good idea, but since there are some space constraints, we need to redesign the application interface which makes this somewhat of a long term change. |
| 1. Make the mouse-over labels on the maps transparent | We have made the mouse-over labels 50% transparent. |
| 1. Arrange the components hierarchically to communicate the order of importance. | It is difficult to decide the appropriate hierarchy because we are not sure which users might find one component more useful than the other. However we decided that certain elements such as the map title should have more prominence and these changes are currently in development. |
| 1. Include a small US map showing state rates to put the atlas map in context | Space constraints make this impossible with our current layout. |
| 1. Ability to change the color palette for both maps and population pyramid. | For the current version of the Atlas we decided not to incorporate this feature. This would require providing another set of user controls. There are always some tradeoffs in terms of the amount of user control we can provide and the amount of complexity that this would add to the interface. |
| 1. Option of turning the county names on the choropleth map | This was difficult given space and placement issues. |
| 1. Express population pyramid data as percent rather than numbers | We decided that numbers were appropriate rather than percentages because the census reports population pyramid data in numbers. |
| 1. Add other types of classification methods, including natural breaks (change the break points in that case) | We discussed this issue and currently our team is incorporating this feature. We also decided that if possible, the most appropriate classification method to include would be the Jenk’s classification method. |
| 1. Have maps with absolute numbers. | Rates are typically considered the appropriate way to report cancer data. |
| 1. Provide the ability to see many maps side by side. | The Atlas currently has the ability to display two maps together. There are space constraints and design considerations which make it difficult to implement this feature. We are planning to incorporate micromaps later and this might help address this issue. |
| 1. Micromap plots | See Discussion section |
| 1. Make the map(s) larger 2. Make the view larger | Space limitations make these requests difficult to address. The atlas is implemented in Flash, so it is possible to stretch the entire layout to fill the screen. |
| 1. Add map projection tools | This is quite complicated and we do not see the need for this for the kind of Atlas we are designing. Most users are novices to GIS tools and as long as we ensure that a standard, appropriate projection has been used, there should be little reason for users to modify it. |
| 1. Undo/step back function | This feature is possible with a substantial rewrite of the Flash interface code, and at this time we have chosen to work on other issues which provide a more immediate return and which were requested by more users. |
| 1. Include Poisson probability estimates, and perhaps Bayesian smoothed map outputs to aid with small numbers problem | Advanced techniques like these are not currently within the scope of the Atlas – which is intended to support public health analysts who do not currently take advantage of much in the way of geographic information. |
| 1. Display red horizontal 95% confidence interval lines (that appear on mouse-over in the graph) as permanent features | We are working on a version of this feature - though it might be designed differently than our users had proposed. |
| 1. Sort the table by any column | This feature has been recently implemented. |
| 1. Moving horizontal/vertical cross-hairs on the frequency plot are distracting at times, especially when a move from one county to another results in a large jump from one point in the distribution to another | Because this was mentioned by a single user and the majority of our users enjoyed the interactive highlighting, we have decided to reserve this issue for the time being. It may be possible in the future to develop a smoother highlighting method. |
| 1. Add the ability to permanently highlight a county (or counties) in the map (perhaps with a red outline or similar) – not just on rollover. | See issue 27. |
| 1. While animation is running allow selection of counties for special attention. | See issue 27. |
| 1. Comparisons of counties (3 suggestions were given for this: either by freezing the UI or the ability to select more than one county or by grouping data) | We are currently implementing this feature. The user would have the ability to press “shift+click” to select more than one county. This however requires updating other features that are linked to the counties. We need to make sure that features such as the population pyramid, the labels for the population pyramid, the data on the table and the frequency plot updates in a reasonable and logical manner. |
| 1. Comparisons of rates to see effects of early detection in different age groups or between two cancer types. | This can be accomplished using the two map option currently built into the Atlas. We are working on the addition of some type of micromap plot that may also serve this purpose in the future. |
| 1. Comparisons of other variables with cancer | This is up to the cooperating agency to provide suitable data – we are able to ingest new data with relative ease. |
| 1. Change levels of geographical aggregation down to zip codes, tracts, or other areal units | CDC provided us with the current data which was aggregated at the county level. We are dependent on CDC about the geographic level at which they are able and willing to provide data. Data at a different geographical level (such as zip code or tracts) may have privacy issues. However for a national level, we are also currently developing another Atlas which is at: <http://www.geovista.psu.edu/grants/national.html>  While we agree that some users may want more geographic detail, that is not possible in a web-based application for the public due to confidentiality constraints. Aggregating up to congressional districts would not create any confidentiality issues, however, there are only 19 congressional districts in Pennsylvania and they cross counties. Thus, creating District-based maps would require going back to the source data to calculate accurate incidence rates by District and the resulting maps would obscure much of the geographic variation in the state. One solution here (that we have not yet implemented) would be to provide the option to superimpose Congressional District boundaries on top of the existing county-based choropleth maps. |
| 1. Provide additional data information on:   - Data source  - Data limitations  - National rates  - Other Metadata  - Patterns and other basic facts on cancer  - Data standardization methods | We are adding a tabbed window in the place where the current table is located. Under several tabs we will have information on the demographic profiles\ of the state and its counties, provide metadata and data standardization information, and offer other details on related sources and facts about cancer. |
| 1. Include risk maps & /or tables, e.g., SES, median income, BRFSS data on smoking, health insurance, obesity, education levels, # or major oncology providers, etc… | The new tabbed window mentioned in issue 28 will have some info on these factors. Otherwise, we are limited to the data we can acquire from our sponsors. |
| 1. Place small images of all of the time-specific maps below the larger map and indicate which of these is being shown – to aid in trend analysis | We are currently investigating ideas like this to help aid temporal analysis. |
| 1. Include a time trend graph | We are investigating strategies to allow users the ability to select one or more counties and view their trends over time. This issue depends in some extent on issue 24. |
| 1. It seems necessary to 'rewind' the animation to be able to play it again | This is a limitation we have experienced developing the Flash interface and we are researching ways to work around it. |
| 1. Clock icon needs improvement | We will be updating the temporal animation icons to make them more obvious. |
| 1. Animation needs some sort of permanent representation -- perhaps line graphs linked back to the static snapshots. This might give a better sense of converging or diverging fortunes in different counties -- increasing/decreasing/stable etc. | We are currently investigating sophisticated ways like this to help aid temporal analysis. |
| 1. Ability to add own data | The final Atlas would be hosted on CDC’s website and adding one’s own data is not a viable option. The Atlas is a prototype for the state of Pennsylvania which is to be used by health departments and cancer registries. |
| 1. Export data | Because the Atlas is going to be hosted on CDC website we must first investigate whether or not CDC will allow the export of data from the Atlas. |
| 1. Obtain data for incidence & mortality by state legislator district | This data is not currently available to us – it would need to be calculated and provided by CDC. |
| 1. In the table it might be useful to show the rates for different time periods -- to make possible trends easier to identify | We are working on visual solutions to the time trend analysis issue – this is one option, but one that we do not prefer because it would be difficult to easily interpret. |
| 1. If the count is an Average, it should say 3 yr av. Count, not just Count | We are working on a solution to clarify this issue in the interface. |
| 1. Add content related to science/evidence based interventions related to the specific cancer type. | We are currently adding this feature as an addition to the tabbed window. |
| 1. Include risk maps & /or tables, e.g., SES, median income, BRFSS data on smoking, health insurance, obesity, education levels, # or major oncology providers, etc ** | We are currently investigating the feasibility of this feature as an addition to the tabbed window. |
| 1. Include a data screen to identify an increase or decrease in stats - or if the data is stable | We are currently investigating the feasibility of this feature as an addition to the tabbed window. |
| 1. Sub-county level of analysis (i.e. census tracts) if feasible | See issue 27. |
| 1. Combined views of rates and absolute counts to get a view of the cancer burden | We are currently investigating the feasibility of this feature. |
| 1. Add to the table: incidence rate Standard Error, rate ratio, rate ratio standard error, rate ratio lower confidence bound, rate ratio upper confidence bound, expected values | We are currently investigating the feasibility of this feature as an addition to the tabbed window. |
| 1. Include state Breast and Cervical Cancer Early Detection and Prevention data (e.g., if program information about number of breast and cervical cancer screening exams). | We are currently investigating the feasibility of this feature as an addition to the tabbed window. |
| 1. Add rate goals set by the counties for those cancer types (“the public would like that”) | We are currently investigating the feasibility of this feature as an addition to the tabbed window. |
| 1. Include age specific rates – for planning purposes how many cases can we expect in the next 10 years based on current rates and population figures | We are currently investigating the feasibility of this feature as an addition to the tabbed window. |
| 1. Include cancer mortality rates | See issue 27 – we can add data like this provided our sponsors can supply it and wish to have it in the Atlas. |
| 1. Include other subsets of population | We are unclear about this issue – since the Atlas already breaks down population by age, race, and stage. |
| 1. Include other cancer sites (e.g., breast, cervical, lung, ovarian, skin, and "total cancer burden" (all cancer combined)) | See issue 27 – we can add data provided our sponsors are able to supply it and are willing to have it in the Atlas. |
| 1. There should be an option to choose a standard x-axis range when displaying one plot and selecting new data (like you can when comparing 2 maps & plots) | We are currently investigating the feasibility of this feature. |
| 1. Allow combining graphs to show two time periods together | We are currently investigating the feasibility of this feature. |
| 1. County-by-county comparisons in bar chart form-allowing selection of several counties vs. state average would be helpful. | The selection feature described in issue 24 will permit this kind of analysis. The state rate is given above the frequency plot and it will be possible to have it display the current selected rates against this average. . |
| 1. Label axes of plot and frequency grouping bar under plot | These additions are currently in development. |
| 1. Integrate age-specific rates into population pyramid (as a suggestion to help average user understand why the pyramid is there). | We are investigating ways in which this has been done in other projects to ensure our implementation will conform to epidemiological norms. |
| 1. When two maps are displayed there may be some confusion over the 'align x-axes' option | We will remove this option when two maps are displayed. |
| 1. Terms: in situ (maybe add in parens "benign" or "not malignant"); quantile - maybe "equal # counties". | A glossary of terms will be added to the tabbed window. |
| 1. State the source of the data, the method of calculating rates and age adjustment of rates | This information will soon be included in the tabbed window. |
| 1. What are the suppression rules? Also, "No data" is not really true - there is data but you've chosen to suppress it for some reason | In the future this information about data constraints will be provided in the tabbed window. |
| 1. Time periods are confusing at first, since the list includes 3 different sets (single year, 3-year rolling, and all years) | We are standardizing this to show only three year averages. |
| 1. Showing significant differences in rates compared the state rate would be helpful for cancer control planning | The PA total and rate is given above the frequency plot. We are working on additional tools to help look for significant changes in rates over time. |
| 1. Provide scatter plot displays of demographic information (say percentage in some population age-sex-race group) against incidence rate. | In its current form it would be very difficult to implement this due to space limitations. We are investigating how we can use micromap plots to accomplish tasks like these. |
| 1. Some sort of Lorenz curve showing summary differences in the spatial distributions between years/sites/subgroups might be useful -- this would be applicable only to the two map display | Advanced techniques like these are not currently within the scope of the Atlas – which is intended to support public health analysts who do not currently take advantage of much in the way of geographic information. |
| 1. Given maps that have been developed so far, what action should be taken (if any)? What are implications for decision making? If possible, for beginner user, it might be helpful if there was a "decision tree" | Decision-modeling is not within the current scope of the Atlas project, but the general idea is perhaps something worth pursuing with a different type of web-based geovisualization tool. |
| 1. Need ways to print, with a pre-formatted print screen containing all tools | Currently we have added a print to file capability and can also print the screen. You can also zoom in to certain elements and also print the screen. |
